# Plasmid Interactions Can Improve Plasmid Persistence in Bacterial Populations

**DOI:** 10.3389/fmicb.2020.02033

**Published:** 2020-08-31

**Authors:** João Alves Gama, Rita Zilhão, Francisco Dionisio

**Affiliations:** ^1^Department of Pharmacy, Faculty of Health Sciences, UiT The Arctic University of Norway, Tromsø, Norway; ^2^Department of Plant Biology, Faculty of Sciences, University of Lisbon, Lisbon, Portugal; ^3^cE3c – Centre for Ecology, Evolution and Environmental Changes, Faculty of Sciences, University of Lisbon, Lisbon, Portugal

**Keywords:** bacteria, plasmids, simulations, epistasis, facilitation, conjugation

## Abstract

It is difficult to understand plasmid maintenance in the absence of selection and theoretical models predict the conditions for plasmid persistence to be limited. Plasmid-associated fitness costs decrease bacterial competitivity, while imperfect partition allows the emergence of plasmid-free cells during cell division. Although plasmid conjugative transfer allows mobility into plasmid-free cells, the rate of such events is generally not high enough to ensure plasmid persistence. Experimental data suggest several factors that may expand the conditions favorable for plasmid maintenance, such as compensatory mutations and accessory genes that allow positive selection. Most of the previous studies focus on bacteria that carry a single plasmid. However, there is increasing evidence that multiple plasmids inhabit the same bacterial population and that interactions between them affect their transmission and persistence. Here, we adapt previous mathematical models to include multiple plasmids and perform computer simulations to study how interactions among them affect plasmid maintenance. We tested the contribution of different plasmid interaction parameters that impact three biological features: host fitness, conjugative transfer and plasmid loss – which affect plasmid persistence. The interaction affecting conjugation was studied in the contexts of intracellular and intercellular interactions, i.e., the plasmids interact when present in the same cell or when in different cells, respectively. First, we tested the effect of each type of interaction alone and concluded that only interactions affecting fitness (epistasis) prevented plasmid extinction. Although not allowing plasmid maintenance, intracellular interactions increasing conjugative efficiencies had a more determinant impact in delaying extinction than the remaining parameters. Then, we allowed multiple interactions between plasmids and concluded that, in a few cases, a combined effect of (intracellular) interactions increasing conjugation and fitness lead to plasmid maintenance. Our results show a hierarchy among these interaction parameters. Those affecting fitness favor plasmid persistence more than those affecting conjugative transfer and lastly plasmid loss. These results suggest that interactions between different plasmids can favor their persistence in bacterial communities.

## Introduction

Mobile plasmids carry genes essential for their replication, conjugative transfer, and stability in the host population (Toussaint and Merlin, 2002). Additionally, they may encode traits useful for their hosts, such as resistance to antibiotics and heavy metals (Foster, 1983), virulence factors (Gyles and Boerlin, 2014) or catabolism of xenobiotics (Nojiri et al., 2004). This genetic cargo promotes bacterial diversification (Heuer and Smalla, 2012) reshaping lifestyles (Kaper et al., 2004; Howard et al., 2009; Hadi, 2020) that can become critical to human populations, as, for instance, the antibiotic resistance crisis (San Millan, 2018).

These traits are beneficial only under specific circumstances and cannot explain plasmid persistence (Carroll and Wong, 2018). Upon cell division, imperfect plasmid segregation allows plasmid-free cells to emerge in the population, and plasmid loss tends to provide a growth advantage in a non-selective environment by removing the plasmid-imposed fitness cost. Moreover, gene migration into the chromosome renders the plasmid obsolete, because plasmid loss would no longer be detrimental to the host even under conditions selecting for beneficial genes since the beneficial gene is kept (Bergstrom et al., 2000).

Although plasmids can enforce their persistence by conjugating back into plasmid-free cells, these rates of transfer seem to be too low to allow long-term plasmid maintenance. The low transfer rates result, at least in part, from plasmid-encoded mechanisms that control the expression of the genes involved in the conjugative process (Frost and Koraimann, 2010). By repressing these genes, for example when there are no plasmid-free cells nearby (Koraimann and Wagner, 2014), the plasmid reduces its conjugation and consequently imposes lower fitness costs on the host (Haft et al., 2009). Furthermore, repression of the conjugative machinery may prevent infection by bacteriophages that use conjugative pili as receptors (Anderson, 1968, but see an alternative explanation in Dionisio, 2005). However, in newly formed transconjugants there is a time-delay until the repression of conjugative genes is re-established. Therefore, plasmids become transitorily derepressed in a community due to these cells, consequently displaying much higher conjugative efficiencies. This effect of transitory derepression that leads to epidemic spread could, in theory, explain plasmid maintenance (Lundquist and Levin, 1986), but the equilibrium density of donor and recipient populations would have to be unrealistically high (Simonsen, 1991).

Mutational events can also increase the odds of plasmid persistence, by reducing the plasmid fitness cost (Dahlberg and Chao, 2003; San Millan et al., 2014b; Harrison et al., 2015; Porse et al., 2016; Stalder et al., 2017) such that sometimes plasmids even become beneficial (Bouma and Lenski, 1988; Dionisio et al., 2005; Starikova et al., 2013; Loftie-Eaton et al., 2017), or improve its stability in the host (Sota et al., 2010; Loftie-Eaton et al., 2016; Yano et al., 2016). Amelioration can occur in a diversity of environments ranging from antagonistic to mutualistic (Harrison et al., 2015), and pulses of positive selection (San Millan et al., 2014b; Stevenson et al., 2018) facilitates plasmid maintenance. Likewise, distinct plasmids affect the host differently (Hall et al., 2015; Di Luca et al., 2017; San Millan et al., 2018) and vice versa, meaning that the same plasmid behaves differently in response to different hosts, varying its fitness/physiological effect (Harr and Schlötterer, 2006; Kottara et al., 2018), conjugative efficiency (Dionisio et al., 2002; De Gelder et al., 2005; Sheppard et al., 2020) and stability (De Gelder et al., 2007). Therefore, plasmid behavior may also differ in populations composed of multiple hosts and/or plasmids (De Gelder et al., 2008; Hall et al., 2016; Kottara et al., 2016; Harrison et al., 2018; Jordt et al., 2020). Indeed, mathematical models that incorporate more complex factors, including fluctuating selective pressures (Peña-Miller et al., 2015), plasmid compensation (Harrison et al., 2016; Hall et al., 2017; Zwanzig et al., 2019), population diversity (i.e., multiple strains) (Dionisio, 2005), and pleiotropy (Jordt et al., 2020) expand the range of conditions that allow plasmid maintenance relatively to earlier models that focused primarily on essential plasmid features (fitness costs, conjugative efficiency, and loss rate) (Stewart and Levin, 1977; Simonsen, 1991; Bergstrom et al., 2000).

Besides experiencing changing environments or the presence of additional strains, bacterial communities may also face several plasmids simultaneously. Indeed, bacteria commonly carry more than one plasmid (San Millan et al., 2014a), which allows interactions among them (reviewed in Dionisio et al., 2019). For example, interactions between plasmids that alter fitness costs (epistasis) (Silva et al., 2011; San Millan et al., 2014a) affect the total cost of harboring the two plasmids simultaneously such that it becomes higher (or lower) than simply the sum of the cost imposed by each plasmid individually. Plasmid behavior may also change whether they occupy the same cells or if they are present in different cells of the same population (Sagai et al., 1977; Gama et al., 2017a). Recently it has also been shown that upon host-plasmid co-evolution under antibiotic selection, the stability of two distinct plasmids in communities consisting of two different bacterial species increased once antibiotics were removed (Jordt et al., 2020). Interestingly, pleiotropic effects resulted in greater plasmid persistence in both novel host-plasmid combinations and, in some cases, multi-plasmid hosts (Jordt et al., 2020).

In this work, we modify earlier mathematical models devoted to studying plasmid maintenance (Stewart and Levin, 1977; Levin et al., 1979; Simonsen, 1991) as to incorporate multiple plasmids and interactions among them. Such interactions affect the three main features of plasmid biology: fitness cost, conjugative efficiency, and rate of loss. Therefore, we aim to understand how interactions between plasmids affect their maintenance in bacterial populations.

## Materials and Methods

### Parameters

To model the ecology of plasmid-harboring bacteria we consider three main parameters of plasmid biology: the fitness effect of plasmid carriage on the host cell, the rate of conjugative transfer and the rate of plasmid loss due to missegregation. These models are adapted from mass-action models by Stewart and Levin (1977), Levin et al. (1979), Simonsen (1991), but we now consider two plasmids X (focal) and Y (competitor). We assume that all cells are isogenic, only differing in plasmid content. We also assume that there is no evolution of hosts or of plasmids, otherwise pleiotropic effects between mutations in different replicons should be considered (Jordt et al., 2020). For language simplification, X may refer to plasmid X, to a cell harboring plasmid X, or even the density of cells harboring plasmid X, depending on the context. The same definitions apply to Y. In a bacterial population where cells carry each of two different conjugative plasmids, there are four possible types of cells: X and Y carrying, respectively, each of the plasmids, XY carrying simultaneously both plasmids as a result of conjugation, and plasmid-free cells Ø produced by plasmid loss. Following the simplification mentioned above, XY may refer to cells harboring both the X and Y plasmids or the density of cells harboring both plasmids, while Ø may refer to plasmid-free cells or their density. The parameters and their values are presented in Table 1.

**TABLE 1:** Parameters used in models.

Symbol^*a*^	Definition	Values^*b*^	Units
Ø	Density of plasmid-free cells	(0)	cells⋅mL^–1^
X	Density of cells carrying (focal) plasmid X	(5 × 10^5^; [10^6^])	cells⋅mL^–1^
Y	Density of cells carrying (competitor) plasmid Y	(0 or 5 × 10^5^)	cells⋅mL^–1^
XY	Density of cells carrying both plasmids X and Y	(0)	cells⋅mL^–1^
R	Resource concentration in the chemostat	(R_0_ = 100)	μg⋅mL^–1^
Ω	Chemostat turnover rate	0.05	h^–1^
Q	Parameter used in Monod function	5	μg⋅mL^–1^
Π	Resource required per cell division	10^–6^	μg
ψ	Standard growth rate	3⋅ln(2)	h^–1^
ω_X_	Fitness of cells carrying plasmid X	0.85; 0.9; 0.95; 0.975; [1; 1.05]	
ω_Y_	Fitness of cells carrying plasmid Y	0.85; {0.9}; 0.95; 0.975	
ω_XY_	Fitness of cells carrying both plasmids X and Y	ω_X_⋅ω_Y_ + ε	
ε	Interaction effect on fitness (epistasis)	−0.05; 0; 0.05; 0.1	
γ_X_	Conjugation rate of plasmid X from cells X to Ø	10^–13^; 10^–12^; 10^–11^; [10^–10^]	mL⋅cell^–1^⋅h^–1^
γ_Y_	Conjugation rate of plasmid Y from cells Y to Ø	10^–13^; {10^–12^}; 10^–11^	mL⋅cell^–1^⋅h^–1^
γ_X(Y)_	Conjugation rate of plasmid X from cells XY to Ø	γ_X_⋅α_X_	mL⋅cell^–1^⋅h^–1^
γ_Y(X)_	Conjugation rate of plasmid Y from cells XY to Ø	γ_Y_⋅α_Y_	mL⋅cell^–1^⋅h^–1^
α	Intracellular interaction effect on the conjugation rate of plasmid X (α_X_) and/or Y (α_Y_)	10^–3^; 1; 10	
γ_X_→_Y_	Conjugation rate of plasmid X from cells X to Y	γ_X_⋅*ξ*_X_	mL⋅cell^–1^⋅h^–1^
γ_Y_→_X_	Conjugation rate of plasmid Y from cells Y to X	γ_Y_⋅*ξ*_Y_	mL⋅cell^–1^⋅h^–1^
ξ	Intercellular interaction effect on the conjugation rate of plasmid X (ξ_X_) and/or Y (ξ_Y_)	10^–2^; 1; 10	
γ_X(Y)_→_Y_	Conjugation rate of plasmid X from cells XY to Y	if α_X_ ≤ 1: γ_X(Y)_; else: γ_X_	
γ_Y(X)_→_X_	Conjugation rate of plasmid Y from cells XY to X	if α_Y_ ≤ 1: γ_Y(X)_; else: γ_Y_	
γ_XY_	Co-transfer rate of plasmids X and Y from cells XY to Ø	Minimum of γ_X(Y)_ and γ_Y(X)_	mL⋅cell^–1^⋅h^–1^
δ	Loss rate of plasmids X or Y from cells X and Y	10^–8^; 10^–6^; 10^–4^	h^–1^
δ_XY_	Loss rate of plasmids X or Y from cells XY	δ⋅σ	h^–1^
σ	Interaction Effect on loss rate	1; 10	

*^a^ω_X_, ω_Y_ and ω_XY_ represent relative fitness, such that the plasmid-free strain Ø has ω_Ø_ = 1. ^b^Values in () represent initialization values, values in [] were used only in control simulations and values in {} were used in simulations where the competitor plasmid had fixed parameters.*

#### Fitness

*Escherichia coli* K-12 has a doubling time of 20 min in Lysogeny Broth (Sezonov et al., 2007), equivalent to three generations per hour, which translates to a standard growth rate ψ = 3⋅ln(2) h^–1^. We assume this to be the maximum growth rate of plasmid-free strains. Since plasmid carriage commonly entails a fitness cost, plasmid-carrying strains will grow at a rate ψ weighed by their fitness ω (relative to the plasmid-free strain), such that the growth rate of a strain carrying plasmid X is ψ_X_ = ψ⋅ω_X_. The fitness cost of different conjugative plasmids has been determined in the ranges 3.9–8% and 0–14.3%, respectively, in *E. coli* (Silva et al., 2011) and *P. aeruginosa* (San Millan et al., 2014a). Accordingly, we employed fitness costs of 2.5%, 5%, 10%, and 15% on the models, translating into ω_X_ (and ω_Y_) ∈ {0.85; 0.9; 0.95; 0.975}. Although other works also report fitness costs for conjugative plasmids, we focused solely on the two above as they also provide another important information for this study, the fitness effects of the interactions (epistasis) between plasmid pairs.

Among conjugative plasmid combinations, positive and negative epistatic interactions (ε) are equally frequent and within a 95% confidence interval of [−0.035; 0.06] (Silva et al., 2011). Epistasis between a conjugative (or a mobilizable) and a non-conjugative plasmid tended to be positive, varying between 0.005 and 0.159 (San Millan et al., 2014a). Therefore, we considered values of epistasis ε∈ {−0.05; 0; 0.05; 0.1}, such that the fitness of strains carrying both plasmids X and Y is ω_XY_ = ω_X_⋅ω_Y_ + ε.

#### Loss

Plasmid-carrying cells can become plasmid-free due to plasmid loss during cell division. This rate of plasmid loss can vary substantially, at least ranging from 10^–9^ to 10^–3^ (Lau et al., 2013; Loftie-Eaton et al., 2017) and we considered it as δ∈ {10^–8^; 10^–6^; 10^–4^}. We considered that plasmids X and Y always have the same loss rate, to reduce the number of possible combinations that would otherwise be too high. Therefore, a proportion δ⋅X of cells carrying plasmid X becomes plasmid-free every hour.

The rate of plasmid loss can be affected by incompatible plasmids that interfere with replication or partition mechanisms (Novick, 1987; Nordström and Austin, 1989), but also disturbed by compatible plasmids that may perturb stability mechanisms such as toxin–antitoxin (also known as post-segregation killing or addiction) systems (Kamruzzaman et al., 2017). Interference between addiction systems can increase plasmid loss at least one order of magnitude (Radnedge et al., 1997), thus we regarded interference on loss rate as σ∈ {1; 10}, such that when the two plasmids are present in the same cell the loss rate for either of them is δ_XY_ = δ⋅σ.

#### Conjugation

The remaining parameter is plasmid transfer which occurs at a specific conjugation rate γ and depends on the proportion of donor (*D*) and recipient (*R*) cells such that transconjugants (*T*) emerge as $\frac{dT}{dt}=\gamma \cdot D\cdot R$. Values for plasmid conjugation rates were based on our previous publication (Gama et al., 2017a). These conjugation efficiencies were converted by the end-point method (Simonsen et al., 1990) to reflect conjugation rates per hour (they are an approximation as our experiments lasted 90 min). Conjugation rates varied between 10^–17^ to 10^–8^. Transfer of plasmids R477-1 (temperature-sensitive transfer) and R6K was near the detection limit as transconjugant colonies were only found occasionally; exclusion of these plasmids revealed a lower bound of 5 × 10^–13^. By contrast, plasmids R1drd19 and F (which are de-repressed for conjugation) and R124 displayed conjugation efficiencies above 10^–10^ characteristic of de-repressed plasmids (Gordon, 1992; Dionisio et al., 2002) and were thus ignored such that their exclusion revealed a higher bound of 5 × 10^–11^. Therefore, we considered conjugation rates γ_X_ (and γ_Y_) ∈ {10^–13^; 10^–12^; 10^–11^} which are within limits previously reported for natural plasmids (Gordon, 1992).

Conjugation rates can be affected by plasmid interactions either if the plasmids reside in the same cell (intracellular effect α) or if one is present in the donor and the other in the recipient cell (intercellular effect ξ). Each of these effects can be negative (inhibiting plasmid transfer) or positive (facilitating plasmid transfer). We calculated these effects by dividing the endpoint conjugation rate of a plasmid when in combination by the respective rate when alone. The median positive intracellular effect was 21.9 and the median negative intracellular effect was 0.006. Therefore, we considered α∈ {10^–3^; 1; 10} as the conjugation rate of X in the presence of Y in the same cell [γ_X(Y__)_] and the conjugation rate of Y in the presence of X [γ_Y__(__X__)_]. The conjugation rate of plasmid X from XY cells to Ø cells (plasmid-free cells) becomes γ_X(Y)_ = γ_X_⋅α_X_ while the conjugation rate of plasmid Y from XY cells to Ø cells becomes γ_Y(X)_ = γ_Y_⋅α_Y_, such that α_X_ and α_Y_ are the respective intracellular effects on X and Y. The median positive intercellular effect was 8.3 while the median negative intercellular effect was 0.04, such that we considered ξ∈ {10^–2^; 1; 10}. The conjugation rate from X cells to Y cells thus becomes γ_X__Y_ = γ_X_⋅ξ_X_ and from Y into X becomes γ_Y__X_ = γ_Y_⋅ξ_Y_, where ξ_X_ and ξ_Y_ are the respective intercellular effects on X and Y.

Three types of transconjugants can develop from matings between cells XY with Ø that may acquire either plasmid X or Y but also acquire both plasmids. Simultaneous transfer of plasmids has been shown to occur at an identical rate as the transfer of the least efficient plasmid in the combination (Gama et al., 2017b), thus we estimated co-transfer γ_XY_ as the minimum between γ_X(Y)_ and γ_Y(X)_. As a consequence of these matings, Ø cells receive both plasmids at a rate γ_XY_ but plasmid X and Y individually at rates γ_X(Y)_ − γ_XY_ and γ_Y(X)_ − γ_XY_, respectively.

Additionally, strains carrying a single plasmid can receive the second one from XY cells. Transconjugants from matings between XY and X cells and between XY and Y cells should occur, respectively, as γ_X(Y)__Y_ = γ_X(Y)_ and γ_Y(X)__X_ = γ_Y(X)_ since they depend on the intracellular interactions between plasmids co-residing in the donor XY cells. However, we consider this to be true only when the intracellular effect decreases the rate of transfer (α ≤ 1). The rationale for this exception is the following. Facilitation occurs because the conjugative pili expressed by the second plasmid help to stabilize the mating pair (Gama et al., 2017a). However, in matings between XY and X cells or between XY and Y cells, one of the plasmids is present in both donor and recipient. Therefore, surface/entry exclusion mechanisms expressed by the plasmid in the recipient cell prevent the same plasmid in the donor cells to transfer efficiently (Garcillan-Barcia and de la Cruz, 2008). Exclusion tends to have a stronger effect on mating efficiency than facilitation (Garcillan-Barcia and de la Cruz, 2008), thus neutralizing it. Therefore, we consider that γ_X(Y)__Y_ = γ_X_ and γ_Y(X)__X_ = γ_Y_ when α > 1.

### General Model

The models employed in this study were adapted from previous works (Stewart and Levin, 1977; Levin et al., 1979; Simonsen, 1991) that describe chemostat cultures. The differential equations 1–5 define the rate of change (derivative in time) of the concentration of nutrients and the density of the cell types.

(1) $$\begin{array}{c} \frac{dØ}{dt}=Ø\cdot \psi \cdot \left(\frac{R}{R+Q}\right)-\text{Ω}\cdot Ø+\delta \cdot \left(X+Y\right)-\\ \;\gamma_{\text{X}}\cdot Ø\cdot X-\gamma_{\text{Y}}\cdot Ø\cdot Y-\\ \;\left(\gamma_{\text{XY}}+\left(\gamma_{X\left(Y\right)}-\gamma_{\text{XY}}\right)+\left(\gamma_{Y\left(X\right)}-\gamma_{\text{XY}}\right)\right)\cdot Ø\cdot XY \end{array}$$

The rate of change of the density of plasmid-free cells at a specific time is given by Eq. (1). The first term defines population growth as the product of population density of plasmid-free cells (Ø) by its growth rate (ψ) and the Monod function $\frac{R}{R+Q}$, which models bacterial growth in a liquid environment relative to the concentration of a limiting nutrient. The second term defines the number of cells that exit the chemostat per volume unit of the chemostat at a washout rate Ω. The third term defines the density of the population of single-plasmid-carrying cells (X or Y) that lose the plasmid at a rate δ. The following terms define the proportion of the density of the population of plasmid-free cells that become transconjugants on different matings, which depends on the rate of conjugation and the population densities of both donor and recipient cells (in this case Ø). The two first conjugation terms describe matings between the plasmid-free recipient cells with donor cells carrying either plasmid X or Y, respectively. The last conjugation term describes matings with donor cells carrying simultaneously both plasmids X and Y, such that three types of transconjugates occur at different rates: γ_XY_ for transconjugants receiving both plasmids, γ_X(Y)_ − γ_XY_ and γ_Y(X)_ − γ_XY_ for those receiving, respectively, only plasmid X and Y.

(2) $$\begin{array}{c} \frac{dX}{dt}=X\cdot \psi \cdot \omega_{\text{X}}\cdot \left(\frac{R}{R+Q}\right)-\text{Ω}\cdot \text{X}-\delta \cdot X+\\ \;\delta_{\text{XY}}\cdot XY+\gamma_{\text{X}}\cdot Ø\cdot X+\left(\gamma_{X\left(Y\right)}-\gamma_{\text{XY}}\right)\cdot \\ \;Ø\cdot XY-\gamma_{Y\to X}\cdot X\cdot Y-\gamma_{Y\left(X\right)\to X}\cdot X\cdot XY \end{array}$$

Equation 2 relates to the population density of cells carrying plasmid X only. The first term corresponding to cell growth is similar to the one in Eq. (1), but takes the product by the strain fitness (ω_X_) to weigh the effect of carrying the plasmid. Second and third terms reflect, respectively, cell washout and loss of plasmid X becoming plasmid-free. Additionally, cells carrying both plasmids can lose plasmid Y at a rate δ_XY_, thus creating cells carrying only plasmid X. The amount of X cells also increases due to cells Ø that receive only plasmid X from matings with either cells X or XY, but decreases due to cells X that receive plasmid Y from matings with either cells Y or XY at rates γ_Y__X_ and γ_Y(X)__X_, respectively.

(3) $$\begin{array}{c} \frac{dY}{dt}=Y\cdot \psi \cdot \omega_{\text{Y}}\cdot \left(\frac{R}{R+Q}\right)-\text{Ω}\cdot \text{Y}-\delta \cdot Y+\\ \;\delta_{\text{XY}}\cdot XY+\gamma_{\text{Y}}\cdot Ø\cdot Y+\left(\gamma_{Y\left(X\right)}-\gamma_{\text{XY}}\right)\cdot \\ \;Ø\cdot XY-\gamma_{X\to Y}\cdot X\cdot Y-\gamma_{X\left(Y\right)\to Y}\cdot Y\cdot XY \end{array}$$

Equation 3 is identical to Eq. (2), but refers to cells carrying only plasmid Y instead.

(4) $$\begin{array}{c} \frac{dXY}{dt}=XY\cdot \psi \cdot \omega_{\text{XY}}\cdot \left(\frac{R}{R+Q}\right)-\text{Ω}\cdot \\ \;\text{XY}-2\cdot \delta_{\text{XY}}\cdot XY+\gamma_{\text{XY}}\cdot Ø\cdot XY+\left(\gamma_{X\to Y}+\gamma_{Y\to X}\right)\cdot \\ \;X\cdot Y+\gamma_{Y\left(X\right)\to X}\cdot X\cdot XY+\gamma_{X\left(Y\right)\to Y}\cdot Y\cdot XY \end{array}$$

Equation 4 describes the kinetics of cells carrying simultaneously both plasmids X and Y and resembles Eqs (2 and 3), except the loss term is doubled (as cells can lose either of the plasmids) and that all conjugation terms lead to an increase in the population density of cells with both plasmids. Note that for simplicity we consider that cells can lose one plasmid at a time, but never simultaneously.

(5) $$\begin{array}{c} \frac{dR}{dt}=\text{Ω}\cdot \left(R_{0}-R\right)-\text{Π}\cdot \psi \cdot \left(\frac{R}{R+Q}\right)\cdot \\ \;\left(Ø+X\cdot \omega_{\text{X}}+Y\cdot \omega_{\text{Y}}+XY\cdot \omega_{\text{XY}}\right) \end{array}$$

Lastly, Eq. (5) describes the kinetics of nutrients in the chemostat, such that at a specific time point their concentration depends on the balance between the amount of nutrients (per volume unit) that enters (Ω⋅*R*_0_) and exits (Ω⋅*R*) the chemostat minus the nutrients exhausted for cell growth. The latter being proportional to the amount of resources required per cell division concentration (Π) and the cumulative growth of each of the different strains.

### Model Implementation

We performed simulations under different models. In model 1, plasmid X was the only plasmid in the population, while in models 2–6 plasmid Y is also present. In model 2, the two plasmids do not interact, while in models 3–6 the plasmids interact according to a single interaction parameter (ε, α, ξ, or σ).

The models were implemented in R (R Core Team, 2018) version 3.4.4 and ran with package rootSolve (Soetaert, 2009) to analyze the steady-state of ordinary differential equations and with package deSolve (Soetaert et al., 2010) for solving the equations across time to find the time required for extinction. Packages doParallel (Microsoft Corporation and Weston, 2019) and foreach (Microsoft and Weston, 2019) were used to run multiple simulations in parallel.

### Analysis

The results obtained for the steady-state are on a continuous scale, such that the decreasing population density can become < 1 without reaching 0 when the population met the stable equilibrium. Thus, we considered the plasmid to go to extinction if there is less than one cell per volume unit (mL, see Table 1) carrying plasmid X (i.e., X < 1 and XY < 1) at the steady-state, otherwise the plasmid is stably maintained which we define here as survival. When the plasmid did not survive, we estimated the time of extinction (ToE) as the first time point (non-negative integer scale) where X < 1 and XY < 1. For model 1, where the populations only carried plasmid X but not Y, these conditions simply become X < 1.

We analyzed the effect of each plasmid interaction (models 3–6) relatively to the “null” models 1 and 2. The analysis against model 1 allows us to understand if the presence of cells carrying a second plasmid Y affects the persistence of the focal plasmid X. For this, we normalized the time of extinction obtained from the model under study by the respective value obtained with model 1. That is, we divided the time of extinction under that model by the respective time of extinction under model 1. After normalization, values > 1 indicate that the plasmid increases its time of extinction, and vice-versa. We quantitatively compared (Kruskal–Wallis or Wilcoxon tests) these values in function of the different parameter values of the interaction to understand how the different parameter values affected the outcome of plasmid X. Next, we did a complementary qualitative analysis. To do so, we categorized the normalized times of extinction as the following outcomes: “increase” for values > 1, “decrease” when < 1 and “null” when = 1. Then we applied χ^2^ tests on the tabulated outcomes per interaction value. We performed equivalent analyses against model 2, which allows us to evaluate how favorable (or not) it is for the focal plasmid X to interact with the co-resident plasmid Y.

Data analyses were performed in R (R Core Team, 2018) version 3.5.1 using the following packages: reshape2 (Wickham, 2007) and data.table (Dowle and Srinivasan, 2019) to handle data structures, heplots (Friendly, 2010) to calculate partial η^2^, FSA (Ogle et al., 2019) and rcompanion (Mangiafico, 2019) for Kruskal–Wallis *post hoc* testing, pscl (Jackman, 2020) and performance (Lüdecke et al., 2020) to calculate logistic regression’s pseudo-*R*^2^. Figures were created using packages ggplot2 (Wickham, 2016), patchwork (Pedersen, 2020), ggthemes (Arnold, 2019), and ggpubr (Kassambara, 2020). Supplementary tables were created using package openxlsx (Schauberger and Walker, 2019).

## Results

### Model 1: One Plasmid Only

We started the study by evaluating the fate of different plasmids when alone in the bacterial population. In this model, the initial population consists of 5 × 10^5^ X cells (as explained in the “Materials and Methods” section, and an X cell is a cell harboring plasmid X). Plasmid loss creates plasmid-free cells, which can later reacquire it through conjugation.

**FIGURE 1:**
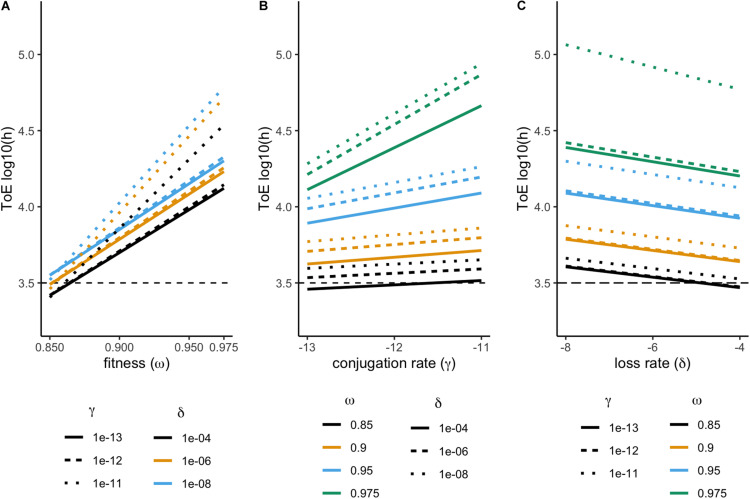
Effect of fitness (ω), conjugation (γ), and loss (δ) rates on the plasmid time of extinction (ToE). Results from model 1, comprising a single plasmid. Time of extinction (ToE) is the first time point where there is less than one plasmid-carrying cell in the population. Time of extinction represented in order to fitness **(A)**, conjugation **(B)**, and loss rate **(C)**.

We studied the fate of 36 plasmids, each consisting of a different combination of features (fitness, conjugation rate, and loss rate). None of these plasmids could be maintained in the chemostat. Therefore, we analyzed their time of extinction (ToE) as a function of the three plasmid features and interactions among them. Time of extinction depends on fitness, conjugation and loss rates but also on the combined effect of fitness and conjugation rate (ANOVA, *df* = 7, *P* < 0.05, Supplementary Table S1). As shown in Figure 1, plasmid-carrying cells take longer to go extinct with increasing fitness and conjugation rates but with decreasing loss rates, as it would be expected. The combined effects of fitness and conjugation rate is illustrated as cells with γ_X_ = 10^–11^ and ω_X_ = 0.975 are not parallel to the others in Figures 1A and B, respectively, while cells with ω_X_ = 0.975 and γ_X_ = 10^–11^ display much higher time of extinction in Figure 1C. This is why, in the ANOVA, the effect size (η^2^) of the fitness-conjugation rate interaction is high – in fact even higher than that of loss (Supplementary Table S1).

### Model 2: Two Non-interacting Plasmids

From now on, we model populations starting with both X and Y cells such that plasmid-free cells will be created by plasmid loss and XY cells by conjugation. In this model 2, as well as the following models 3 to 6, we simulated populations starting with 5 × 10^5^ X cells and 5 × 10^5^ Y cells, such that the initial population size is 10^6^ cells. To control for this change, we repeated the simulation of a single plasmid (model 1) considering the initial population as 10^6^ X cells instead of 5 × 10^5^ X cells. The result is qualitatively the same since no plasmid survives (i.e., no plasmid was stably maintained), while, quantitatively, the time of extinction only increased by 1 h or remained unchanged.

In model 2, although all mating combinations are possible, the interactions between plasmids have no effect (ε = 0 and α = ξ = σ = 1). Both focal (X) and competitor (Y) plasmids express all 36 feature combinations, which consequently results in 432 combinations of two plasmids. In all cases the focal plasmid X always becomes extinct, as well as plasmid Y. In 36 cases the two plasmids become extinct at the same time, which is when they display the same characteristics, while in 198 cases plasmid X disappears before Y. Among those 198 cases, plasmid X becomes extinct faster than plasmid Y when imposing higher fitness cost or when having the same fitness effect but lower conjugation rate (note that plasmids X and Y always have identical loss rates). In the remaining 198 cases, Y cells disappear before X cells, since plasmid features are inverted.

**FIGURE 2:**
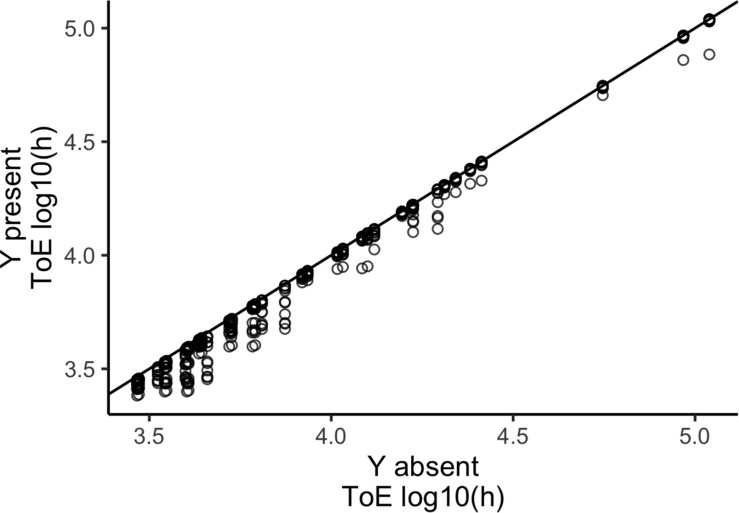
A competitor plasmid Y accelerates the extinction of the focal plasmid X. The vertical axis represents the time of extinction (ToE) of plasmid X when alone in the bacterial population (model 1) and the horizontal axis represents ToE of plasmid X when a competitor plasmid Y is present in the same population (model 2). The blackline represents *y* = *x*.

We compared the times of extinction for plasmid X obtained from models 1 and 2 to understand if the presence of cells carrying a second plasmid Y affects its persistence (Figure 2). When a second plasmid is present the time of extinction decreases (paired Wilcoxon test with continuity correction, *P* < 2.2 × 10^–16^). This shows that competition with another plasmid-carrying strain accelerates plasmid extinction.

### Model 3: Interactions Affecting Fitness (Epistasis)

In model 3, the two plasmids can interact, affecting the total fitness effect on XY cells. Thus, ω_XY_ = ω_X_⋅ω_Y_ + ε, where ε∈ {−0.05; 0; 0.05; 0.1}. This data set consists of a total of 1728 cases, 432 per value of ε.

#### Plasmid Survival

The focal plasmid can survive in 128 of these cases, 105 where epistasis is positive with ε = 0.1 and 23 cases if ε = 0.05. These positive values of epistasis imply that, in all these cases, the fitness of XY cells is higher than that of plasmid-free cells and they are always present in the final population at higher frequencies than X or Y cells. Interestingly, in 45 cases there are no plasmid-free cells in the final population. This happens when loss rates are very low, namely δ = 10^–8^. In the remaining 83 cases, the maximum frequency of plasmid-free cells is 18.66%.

Yet, there are seven more cases where the fitness of cells carrying both plasmids is higher than that of plasmid-free cells (ω_XY_ ≥ 1) but the focal plasmid goes to extinction. All these exceptions have loss rate δ = 10^–4^, four consisting in positive epistasis of ε = 0.05 and ω_X_ = ω_Y_ = 0.975 and the remaining three in ε = 0.1 and ω_X_ = ω_Y_ = 0.95. Additionally, in these seven cases, γ_X_ and γ_Y_ < 10^–11^ (but not γ_X_ = γ_Y_ = 10^–12^ when ε = 0.1). These results show that the plasmids can survive if there is positive epistasis, elevating the fitness XY cells above that of the plasmid-free cells. Nonetheless, there is no guarantee of plasmid survival as lower conjugation rates preclude the emergence of XY cells and higher loss rates decrease their maintenance.

As just mentioned, in 135 cases carriage of two plasmids confers a fitness benefit to the host cells relatively to plasmid-free cells. Despite that, in model 1, we only considered plasmids that produce fitness costs. As a control, we expanded model 1 with ω_X_ ≥ 1, such that the focal plasmid confers no fitness cost or even a benefit (thus ω_X_ ∈ {1; 1.05}). We considered a fitness benefit of 5% because, when XY cells had a fitness higher than that of plasmid-free cells, the fitness effect ranged between 0.000625 and 0.050625. Among the new 18 simulated cases, there are two where plasmid X goes extinct – when ω_X_ = 1 and γ_X_ < 10^–11^ and δ = 10^–4^. These two cases of extinction further illustrate the conceivable detrimental effect of conjugation and loss rates. Moreover, even in the cases where the plasmid can be maintained, the population at equilibrium is not pure and plasmid-free cells exist at a frequency varying from 2.96 × 10^–6^ to 1 × 10^–1^. Overall, this result shows that fitness alone is not sufficient for plasmid survival and depends on the rates of conjugation and loss. It is also interesting to note that plasmid-free cells still persist in these conditions, unlike what was observed for several cases when another plasmid is present in the population.

#### Time of Extinction

Among the 1600 cases where the focal plasmid does not survive there are 736 cases of positive epistasis. In 135 of these cases, XY cells are still less fit than both X and Y cells and are the first to get extinct. In 223 cases XY cells are fitter than both X and Y, but they are the ones displaying the longest time of extinction in only 58 cases. This again shows that XY cells are not necessarily stable even if they are fitter than X and Y. Therefore, even when there is positive epistasis between plasmids, conjugation and loss rates are still determinant for plasmid maintenance.

Among the 432 cases of negative epistasis, XY cells are always less fit than either X or Y cells. Nonetheless, in 12 cases, XY cells are not the first getting extinct – if γ_X_ = γ_Y_ = 10^–11^ and δ > 10^–4^ and either ω_X_ or ω_Y_ (but not both) is 0.975. Thus, provided that both plasmids exhibit high conjugation and low loss rates and one of them imposes low fitness costs XY cells can persist longer that one of the single-plasmid-carrying strains.

**FIGURE 3:**
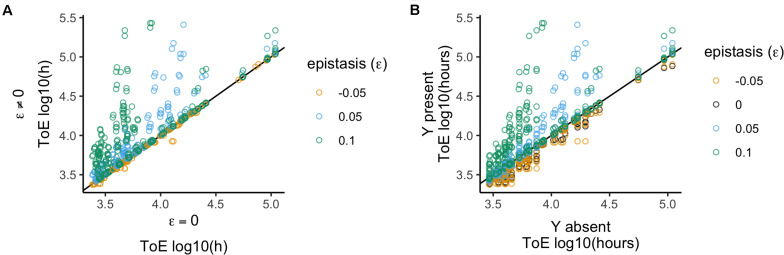
Relative time of extinction when plasmid interactions affect fitness (epistasis; ε). **(A)** Relative time of extinction (ToE) in presence and absence of epistasis: the vertical axis represents ToE of plasmid X when there is epistasis (model 3) and the horizontal axis represents the respective ToE when there are no plasmid interactions (ε = 0; model 2). **(B)** Relative ToE in presence and absence of a competitor plasmid Y: the vertical axis represents ToE of plasmid X when Y is present and plasmid interactions affect fitness (model 3) while the horizontal axis represents the respective ToE when plasmid X is alone in the bacterial population (model 1). The analysis is always in function of the focal plasmid X. The blackline represents *y* = *x*.

We compared these results with those obtained with model 2 to unveil how epistatic interactions affect the fate of the focal plasmid relative to when the two plasmids did not interact (Figure 3A). Therefore, we analyzed the 1600 times of extinction after normalizing them by the results of model 2 – which is equivalent to divide by the respective value when there was no epistasis (ε = 0).

We analyzed the normalized data in function of the different values of epistasis. This type of interaction has a significant effect (Kruskal–Wallis test, *df* = 3, *P* < 2.2 × 10^–16^, Supplementary Table S2), and all four epistasis values produce distinct results (Dunn test with Bonferroni correction, Supplementary Table S2). We further analyzed the effects qualitatively, i.e., we checked if times of extinction increased, decreased or did not change. These results show that the four epistasis values produce different results (χ^2^ test with Yates continuity correction, *df* = 6, *P* < 2.2 × 10^–16^ followed by Fisher pairwise tests of independence, Supplementary Table S2), which agrees with the conclusion of the quantitative analysis. Altogether the results correspond to the prediction that the times of extinction increase with the strength of positive epistasis and decrease with negative epistasis. However, it is important to mention that it is not straightforward to conclude the outcome based on epistasis only because the result also depends on the features of the two plasmids, namely fitness impact, conjugative transfer and loss rate. This means that with positive epistasis not all cases reveal an increased time of extinction, and the reverse is also true for negative epistasis. In addition, ε = −0.05 (when normalized relatively to ε = 0) has a much smaller effect (median = 1 and standard deviation = 0.03) than ε = 0.05 (median 1 and standard deviation = 1.25), showing that negative epistasis has a less detrimental effect than positive epistasis has a beneficial effect.

We also analyzed the 1600 cases to understand how the times of extinction differ from when the competitor plasmid is absent (Figure 3B), that is, we compared them with those obtained with model 1. We analyzed this effect by normalizing the time of extinction of the focal plasmids by the respective value obtained when they were alone in the population (model 1). Positive epistasis has a significant effect (Kruskal–Wallis test, *df* = 3, *P* < 2.2 × 10^–16^, followed by *post hoc* Dunn test with Bonferroni correction, Supplementary Table S3), such that there is a stronger effect for ε = 0.1 and then for ε = 0.05; but ε = −0.05 and ε = 0 do not differ from each other. We obtained the same conclusion when analyzing the effects qualitatively (χ^2^ test with Yates continuity correction, *df* = 3, *P* < 2.2 × 10^–16^ followed by Fisher pairwise tests of independence, Supplementary Table S3), i.e., comparing the number of cases among the categories where times of extinction increase, decrease or do not change. Thus, negative epistasis does not lead to a significantly different outcome than not having epistasis, and the plasmid always gets extinct earlier than when alone. Nevertheless, the number of cases of increased plasmid persistence increases with the strength of positive epistasis (Supplementary Table S3).

### Model 4: Intracellular Interactions Affecting Conjugation

In this model, the two plasmids can interact, such that the conjugation rate of one of the plasmids changes when both plasmids occupy the same cell – an intracellular conjugation effect α. There are two possibilities: either X or Y is the target of the effect, but never both. Thus, either the transfer rate of X when co-inhabiting with Y is the transfer rate of X when alone times α_X_ (γ_X(Y)_ = γ_X_⋅α_X_) or the transfer rate of Y when co-inhabiting with X is the transfer rate of Y when alone times α_Y_ γ_Y(X)_ = γ_Y_⋅α_Y_), where α_X_ or α_Y_ ∈ {10^–3^; 1; 10}. Next, we analyze both possibilities, but always in function of plasmid X. Each of these data sets consists of a total of 1296 cases, 432 per value of the intracellular conjugation effect α.

#### Plasmid X as the Target of Interactions

**FIGURE 4:**
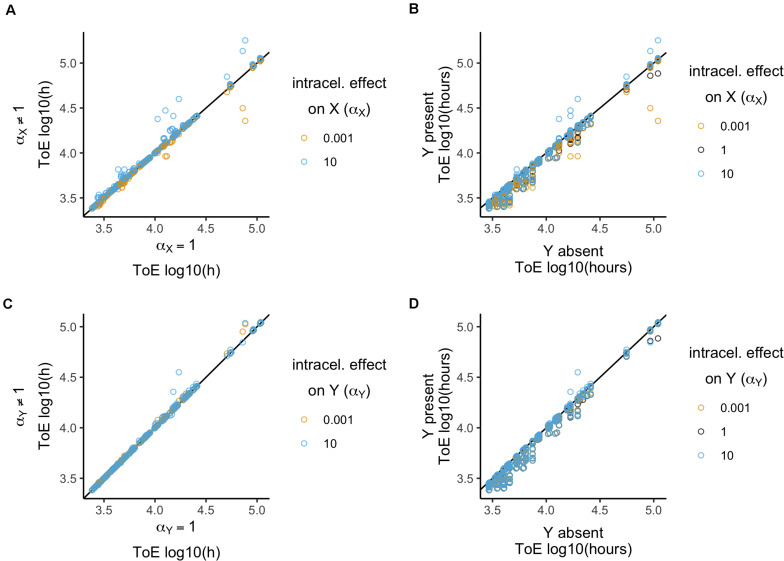
Relative time of extinction when plasmid intracellular interactions (α) affect conjugation rates. **(A,C)** Relative time of extinction (ToE) in presence and absence of interactions: the vertical axis represents ToE of plasmid X when the plasmids interact (model 4) and the horizontal axis represents the respective ToE when the plasmids do not interact (α = 1; model 2). **(B,D)** Relative ToE in presence and absence of a competitor plasmid Y: the vertical axis represents ToE of plasmid X when Y is present and plasmid intracellular interactions affect conjugation rates (model 4) while the horizontal axis represents the respective ToE when plasmid X is alone in the bacterial population (model 1). In panels **(A,B)**, plasmid X is the target of the effect α_X_, while in panels **(C,D)** plasmid Y is the target of effect α_Y_ (models 4.1 and 4.2, respectively). The analysis is always in function of the focal plasmid X. The blackline represents *y* = *x*.

The focal plasmid cannot survive in any of the 1296 cases when it was the target of intracellular conjugation effects. Therefore, we analyzed the times of extinction, comparing among the three values of the intracellular conjugation effect α_X_ (Figure 4A). These values were normalized by the respective values when α_X_ = 1 (which is equivalent to divide by the respective values of model 2). There is a significant effect (Kruskal–Wallis test, *df* = 2, *P* < 2.2 × 10^–16^, Supplementary Table S4) revealing differences between the three α_X_ parameter values (Dunn test with Bonferroni correction, Supplementary Table S4). Even though conjugation rate increases only 10 times, it has a stronger impact (median = 1, standard deviation = 0.14) than when it decreases 1000 times (median = 1, standard deviation = 0.05). We further analyzed the effects qualitatively, i.e., whether times of extinction increased, decreased or did not change. The three α_X_ parameter values produce different results (χ^2^ test with Yates continuity correction, *df* = 4, *P* < 2.2 × 10^–16^ followed by Fisher pairwise tests of independence, Supplementary Table S4), which agrees with the quantitative analysis. While with α_X_ = 10^–3^ times of extinction either decrease (198 cases) or remain the same, with α_X_ = 10 they increased in 247 cases but never decreased.

Next, we compared how times of extinction differ from those obtained with model 1 when the competitor plasmid was absent (Figure 4B). Only α_X_ = 10 has a significant effect (Kruskal–Wallis test, *df* = 2, *P* < 1.3 × 10^–6^, followed by *post hoc* Dunn test with Bonferroni correction, Supplementary Table S5), while α_X_ = 10^–3^ and α_X_ = 1 are not significantly different. Then, we analyzed the effects qualitatively. While all cases with α_X_ = 10^–3^ and α_X_ = 1 the time of extinction decreased relatively to model 1, α_X_ = 10 led to increase in 46 cases (χ^2^ test with Yates continuity correction, *df* = 2, *P* < 2.2 × 10^–16^ followed by Fisher pairwise tests of independence, Supplementary Table S5). In all these 46 cases, the focal plasmid had γ_X_ = 10^–11^ and α_X_ = 10 such that in cells carrying both plasmids γ_X(Y)_ = 10^–10^. Therefore, we tested the outcome of plasmids with γ_X_ = 10^–10^ when alone in a population (model 1). This revealed that all 12 plasmids could be stably maintained in the population, such that the frequencies of plasmid-free cells would vary between 1.15 × 10^–6^ and 7.8 × 10^–2^.

In conclusion, only an increasing intracellular effect on conjugation affected times of extinction significantly, although this was not sufficient to allow plasmid survival in the presence of a second plasmid.

#### Plasmid Y as the Target of Interactions

When the competitor plasmid was the target of intracellular conjugation effects the focal plasmid could not survive in any of the 1296 cases. We analyzed the time of extinction of the focal plasmid X in terms of the intracellular conjugation effect α_Y_ (Figure 4C) relative to model 2. There is a significant effect and the three α_Y_ parameter values produce significantly different results (Kruskal–Wallis test, *df* = 2, *P* < 2.2 × 10^–16^, followed by Dunn test with Bonferroni correction, Supplementary Table S6). There is also a significant difference when analyzing the effects qualitatively (χ^2^ test with Yates continuity correction, *df* = 4, *P* < 2.2 × 10^–16^ followed by Fisher pairwise tests of independence, Supplementary Table S6). The time of extinction of plasmid X increased in 211 cases when α_Y_ = 10^–3^ but in 187 when α_Y_ = 10. However, there are also cases in which the time of extinction decreased: 3 cases when α_Y_ = 10^–3^ and 65 when α_Y_ = 10. Interestingly, the quantitative and qualitative analyses reveal different aspects of these results. Both results with α_Y_ = 10^–3^ and α_Y_ = 10 have a median of 1. Though α_Y_ = 10 allows the time of extinction to increase in fewer cases, it has a stronger effect in the sense that it produces a higher standard deviation (0.06 vs. 0.02). Nonetheless, unlike the previous section, the survival of the focal plasmid can increase whether the effect is below or over 1.

There was no significant difference related to the effect α_Y_ (Kruskal–Wallis test, *df* = 2, *P* = 0.06, Supplementary Table S7) when analyzing times of extinction relative to model 1 where the competitor plasmid was absent (Figure 4D). However, times of extinction always decreased with the exception of 21 cases with α_Y_ = 10 which were sufficient to produce a significant difference (χ^2^ test with Yates continuity correction, *df* = 2, *P* = 5.37 × 10^–10^ followed by Fisher pairwise tests of independence, Supplementary Table S7).

### Model 5: Intercellular Interactions Affecting Conjugation

As in model 4, the two plasmids can interact but the conjugation rate of only one of them changes when both donor and recipient cells carry plasmids – an intercellular conjugation effect ξ. Here there are again two non-overlapping possibilities: either X or Y is the target of the effect, that we analyze only in function of the focal plasmid X. Thus, either γ_X_→_Y_ = γ_X_⋅ξ_X_ or γ_Y_→_X_ = γ_Y_⋅ξ_Y_, where ξ∈ {10^–2^; 1; 10}. Each of these data sets consists on a total of 1296 cases, 432 per value of *ξ*.

#### Plasmid X as the Target of Interactions

**FIGURE 5:**
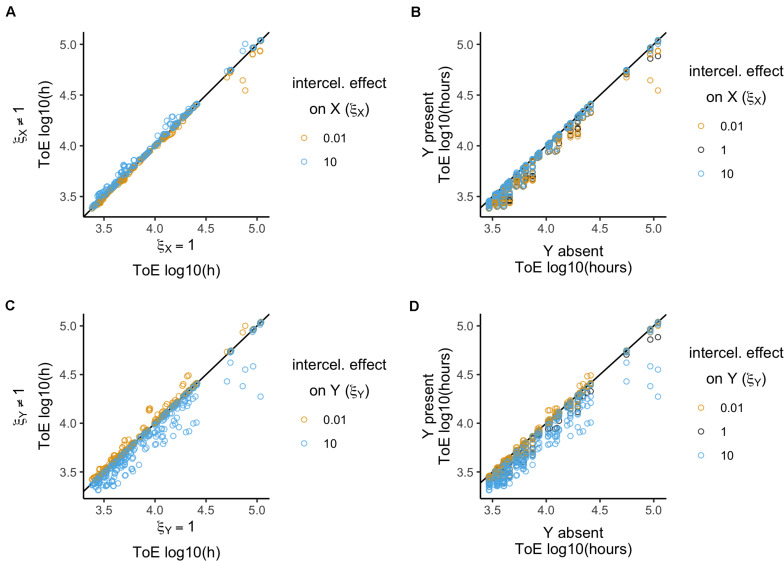
Relative time of extinction (ToE) when plasmid intercellular interactions (ξ) affect conjugation rates. **(A,C)** Relative time of extinction (ToE) in presence and absence of interactions: the vertical axis represents ToE of plasmid X when the plasmids interact (model 5) and the horizontal axis represents the respective ToE when the plasmids do not interact (ξ = 1; model 2). **(B,D)** Relative ToE in presence and absence of a competitor plasmid Y: the vertical axis represents ToE of plasmid X when Y is present and plasmid intercellular interactions affect conjugation rates (model 5) while the horizontal axis represents the respective ToE when plasmid X is alone in the bacterial population (model 1). In **(A,B)** plasmid X is the target of the effect ξX while in **(C,D)** plasmid Y is the target of effect ξY (models 5.1 and 5.2 respectively). The analysis is always in function of the focal plasmid X. The blackline represents *y* = *x*.

The focal plasmid does not persist in any of the 1296 cases where it was the target of intercellular conjugation effects. We normalized times of extinction relatively to ξ_X_ = 1 (model 2) to evaluate the effect of the different values of ξ_X_ (Figure 5A). The three values of ξ_X_ produce significantly different results (Kruskal–Wallis test, *df* = 2, *P* < 2.2 × 10^–16^, followed by Dunn test with Bonferroni correction, Supplementary Table S8). An increase of conjugation rate with ξ_X_ = 10 displays a slightly stronger impact (median = 1, standard deviation = 0.06) than a decrease with ξ_X_ = 0.01 (median = 1, standard deviation = 0.04). Moreover, with ξ_X_ = 0.01 the time of extinction either decreases (313 cases) or remains the same while with ξ_X_ = 10 the time of extinction increased in most cases (408/432) and did not change in the remaining 24 (in all of which γ_X_ = 10^–13^). This difference in outcomes is significant (χ^2^ test with Yates continuity correction, *df* = 4, *P* < 2.2 × 10^–16^ followed by Fisher pairwise tests of independence, Supplementary Table S8).

In Figure 5B, we show how times of extinction differ from when the focal plasmid is alone in the population (model 1). There is a significant difference (Kruskal–Wallis test, *df* = 2, *P* = 8.2 × 10^–7^, followed by *post hoc* Dunn test with Bonferroni correction, Supplementary Table S9), such that ξ_X_ = 10 displays a higher median. On the other hand, a qualitative analysis revealed no significant differences (χ^2^ test with Yates continuity correction, *df* = 2, *P* = 0.05) such that the time of extinction always decreases, except in three cases (where ω_X_ = 0.9, γ_X_ = 10^–11^, δ < 10^–8^ and ξ_X_ = 10).

In conclusion, an increasing intercellular conjugation effect can increase the time of extinction but with a very limited impact and still not allowing plasmid maintenance.

#### Plasmid Y as the Target of Interactions

The focal plasmid could not survive in any of the 1296 cases when the competitor plasmid was the target of intercellular conjugation effects. Figure 5C displays times of extinction in terms of the intercellular conjugation effect ξ_Y_. There is a significant effect and the three ξ_Y_ parameter values produce significantly different results (Kruskal–Wallis test, *df* = 2, *P* < 2.2 × 10^–16^, followed by Dunn test with Bonferroni correction, Supplementary Table S10). However, times of extinction of plasmid X tend to increase when ξ_Y_ < 1 and to decrease when ξ_Y_ > 1, which is the opposite outcome of when plasmid X is the target of interaction instead of Y. Therefore, preventing the acquisition of plasmid Y reduces the rate of formation of less fit XY cells, that would otherwise accelerate extinction of plasmid X as they are more easily outcompeted in the population. When analyzed qualitatively ξ_Y_ > 1 leads to a decrease in all cases while ξ_Y_ < 1 increases times of extinction in 393 out of 432 cases (χ^2^ test with Yates continuity correction, *df* = 4, *P* < 2.2 × 10^–16^, followed by Fisher pairwise tests of independence, Supplementary Table S10).

Next, we compared times of extinction with those from model 1, which lacks the competitor plasmid (Figure 5D). Unlike when plasmid X was itself the target of the intercellular interaction, there are significant effects if plasmid Y is the target instead (Kruskal–Wallis test, *df* = 2, *P* < 2.2 × 10^–16^, Supplementary Table S11). This shows that if X cells acquired plasmid Y at lower rates then plasmid X can be maintained for longer times in the population, while if acquired at higher rates plasmid Y will have a detrimental effect on plasmid X’s persistence. The qualitative analysis reveals the same conclusion (χ^2^ test with Yates continuity correction, *df* = 2, *P* = 3 × 10^–12^ followed by Fisher pairwise tests of independence, Supplementary Table S11). With ξ_Y_ = 0.01 there are 26 cases where ToE increased, all of which have γ_Y_ = 10^–11^. For the other values of ξ_Y_ tested, times of extinction decreased in all cases.

Therefore, plasmid X can persist longer if it reduces the rate of acquiring plasmid Y.

### Model 6: Interactions Affecting Loss

**FIGURE 6:**
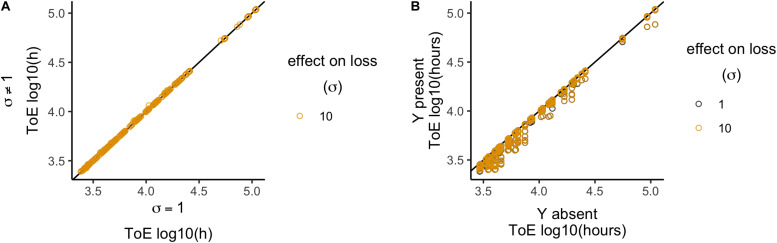
Relative time of extinction (ToE) when plasmid interactions (δ) affect loss rates. **(A)** Relative time of extinction (ToE) in presence and absence of interactions affecting loss rates: the vertical axis represents ToE of plasmid X when there are interactions (model 6) and the horizontal axis represents the respective ToE when there are no interactions (σ = 1; model 2). **(B)** Relative ToE in presence and absence of a competitor plasmid Y: the vertical axis represents ToE of plasmid X when Y is present and plasmid interactions affect loss rates (model 6) while the horizontal axis represents the respective ToE when plasmid X is alone in the bacterial population (model 1). The analysis is always in function of the focal plasmid X. The blackline represents *y* = *x*.

In this model, the plasmid interactions lead to increased loss rates, such that δ_XY_ = δ⋅σ, where σ∈ {1; 10}. This data set consists on 864 total cases, 432 per value of σ. The plasmid cannot survive in any of these cases, so we only analyzed the times of extinction. When normalized relatively to model 2, the times of extinction for plasmid X can vary significantly (Wilcoxon test, *P* < 2.2 × 10^–16^, Supplementary Table S12) but with little deviation (median = 1, standard deviation = 7.6 × 10^–3^; Figure 6A). Times of extinction increased in 170 cases, decreased in 15 (all when ω_*y*_ = 0.975 and σ = 10) and did not change in 247, thus being significantly different from when the plasmids did not interact, i.e., σ = 1 (χ^2^ test with Yates continuity correction, *df* = 2, *P* = 2.2 × 10^–16^). When compared relatively to the condition when plasmid X is alone in the population (model 1), both σ = 1 and σ = 10 decreased the times of extinction (Figure 6B). Nonetheless, the outcomes of σ = 1 and σ = 10 are significantly different (Wilcoxon test, *P* < 2.2 × 10^–16^, Supplementary Table S13), but the effect is very small (σ = 1, median = 9.66 × 10^–1^, standard deviation = 8.87 × 10^–2^; σ = 10, median = 9.67 × 10^–1^, standard deviation = 8.83 × 10^–2^). In conclusion, this interaction has a very limited effect on the time of extinction.

### Multiple Interactions

**FIGURE 7:**
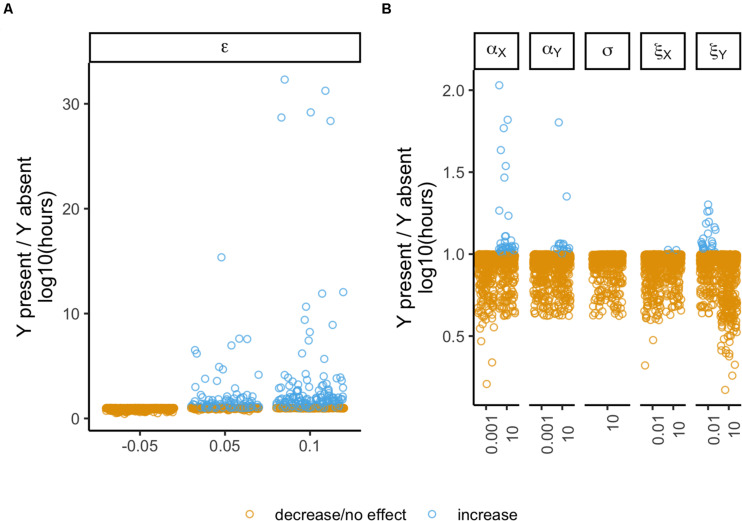
Outcome of the different plasmid interactions. The vertical axis represents the ratio of time of extinction (ToE) when the plasmids interact (models 3–6) by the respective ToE of when the focal plasmid is alone in the population (model 1). The horizontal axis represents the different values of the variable annotated on the top facet. Epistasis (ε) and the remaining parameters are split in different panels to facilitate visualization of the points. Panel **(A)** represents the effect of epistasis (ε) while panel **(B)** refers respectively to the following parameters: intracellular interactions affecting the conjugative efficiency of plasmid X (α_X_) and of plasmid Y (α_Y_), interactions affecting loss rates (δ) and, intercellular interactions affecting the conjugative efficiency of plasmid X (ξ_X_) and of plasmid Y (ξ_Y_). Blue dots represent plasmid combinations where the interactions increase ToE relatively to when the plasmid was alone in the population, while yellow dots represent no change or a decrease in ToE.

Our ultimate goal is to understand if the presence of a second plasmid can be beneficial, and which type of interactions are determinant to prevent/delay extinction. To attain this goal, we collected the results obtained with the models where both plasmids are present, including only those cases where there were interactions. From these 5184 unique cases, the focal plasmid went to extinction in 5056. We normalized their times of extinction relative to the same focal plasmid when alone (model 1), such that the plasmid benefits from a second plasmid if the ratio is greater than one. There was a total of 371 cases where there was a benefit. Then we analyzed the proportion of outcomes (benefit vs. no benefit) among the different variables, which retrieved a significant result (χ^2^ test with Yates continuity correction, *df* = 5, *P* < 2.2 × 10^–16^, followed by Fisher pairwise tests of independence, Supplementary Table S14) showing that some interactions have stronger impacts than others (Figure 7). Epistatic interactions have the strongest effect with 23.54% cases of benefit – in these cases, the focal persists longer in the presence of the second plasmid than when there is no other plasmid in the population. The intracellular effect on plasmid X follows with 5.32% cases of benefit, thus being the second most important plasmid-interacting allowing plasmid persistence to increase. Then follows the conjugation effects on plasmid Y (which do not differ significantly), respectively, intracellular and intercellular effects with 2.43% and 3%. The intercellular effect on plasmid X and the effect on the rate of loss (not differing from each other) follow, respectively, with 0.35% and 0%, thus having the least determinant role on plasmid persistence. The important impact of epistasis is further highlighted as this was the only interaction allowing the focal plasmid to persist in 128 cases (of model 3).

To further understand the contribution of the different interactions to plasmid maintenance we simulated cases where multiple interactions may occur, thus getting all possible combinations from no interactions at all to all interactions happening simultaneously. To avoid an overwhelming number of cases, we considered a single competitor plasmid Y with ω_Y_ = 0.9 and γ_Y_ = 10^–12^. Note that in the previous sections none of the combinations involving this set of Y plasmids could survive nor could the fitness of XY cells be higher than that of plasmid-free cells (ω_XY_ ≥ 1). Among these 23328 simulations, plasmid X could survive in 291 (1.25%) existing in both X and XY cells. These cases share the following features: ε = 0.1, α_X_ = 10, γ_X_ = 10^–11^ and ω_X_ > 0.85. This again suggests that epistasis and the intracellular conjugation effect on plasmid X are the interactions most determinant for plasmid fate. Interestingly, plasmid Y can survive in the same 291 cases, where Y cells constitute at most a proportion of 9.47% of the population. Plasmid-free cells are absent in 144 of these 291 cases – only when δ = 10^–8^.

We performed a logistic regression on the 23037 cases of extinction considering the following interactions (as continuous variables): ε, α_X_, α_Y_, ξ_X_, ξ_Y_ and σ. The independent variable comprises two categories: “increase” with 3222 cases where the time of extinction increases relatively to when the plasmid is alone in the population and “decrease/null” for the remaining 19815 cases. The results are shown in Supplementary Table S15. All the six interactions have significant effects (*P* < 0.05, 34.72% ≤ pseudo *R*^2^ ≤ 47.05%, Supplementary Table S15). As suggested above, ε and α_X_ display more determinant effects (higher deviance from the null model and higher individual pseudo *R*^2^), while σ has the lowest effect on delaying extinction. We repeated this analysis now including the 291 cases of survival as “increase” and the result is qualitatively similar (*P* < 0.05, 38.07% ≤ pseudo *R*^2^ ≤ 50.09%, Supplementary Table S16). Lastly, we analyzed a subset of cases consisting only on the conditions that allow plasmid survival, i.e., all cases where ε = 0.1, α_X_ = 10, γ_X_ = 10^–11^ and ω_X_ > 0.85. This subset has 291 cases categorized as “survival” and 195 as “extinction.” Only α_Y_, ξ_X_, ξ_Y_ and σ were used as predictors for the logistic regression since ε and α_X_ display no variance in this subset. The result shows that only ξ_X_ and ξ_Y_ are significant (*P* < 0.05, 6.95% ≤ pseudo *R*^2^ ≤ 9.23%, Supplementary Table S17). Therefore, we can conclude that these four interactions alone have a weaker impact on the outcome than epistasis and intracellular interactions (ε and α_X_).

## Discussion

It is crucial to understand the conditions favoring plasmid maintenance in natural communities, which can vary in complexity with an increasing number of hosts and plasmids occupying the same ecological habitat. This work aimed to evaluate which plasmid properties and interactions mostly affect the time of persistence in the bacterial population. Our initial results show that plasmids cannot persist in bacterial populations unless they provide a fitness advantage (strictly mutualistic) or have high conjugation rates (strictly parasitic), which supports previous works (Stewart and Levin, 1977; Simonsen, 1991). Expanding these models by allowing bacterial populations to carry an additional non-interactive plasmid reveals, as expected, that plasmid persistence decreases due to competition between plasmids. Our main aim was to examine the role of different plasmid interactions, namely affecting host fitness, conjugative efficiency and the rate of loss due to missegregation, on plasmid persistence. We conclude that although all interactions evaluated can affect plasmid persistence, there is a hierarchy such that interactions affecting fitness (epistasis) have a stronger impact on plasmid maintenance than those affecting conjugation, and lastly plasmid loss.

If plasmids display epistasis and interact simultaneously at no other level, they can be maintained stably in the population if their combined effects provide a fitness benefit to their host cells. Interestingly, in some cases, plasmid-free cells may even be absent from the final population. This, yet, was never observed when populations started only with a single (beneficial) plasmid. However, fitness alone does not ensure plasmid persistence, such that beneficial plasmids or plasmid combinations can still get extinct. Indeed, our results show that plasmid combinations with positive epistasis could only persist if associated with low fitness costs, high conjugation rates and low loss rates. Nonetheless, this situation might not be entirely realistic because the expression of conjugative traits is subject to fitness costs due consumption of energy/metabolites – resulting in a tradeoff such that plasmids may enjoy high conjugative transfers or low cost, but not both (Turner et al., 1998; Porse et al., 2016). In addition, conjugation may render plasmid-carrying cells susceptible to infection by the so-called male-specific phages (phages that only infect cells expressing sex-pili), which decreases their persistence (Jalasvuori et al., 2011; Ojala et al., 2013).

When plasmid interactions could affect multiple parameters (fitness, conjugation, loss) simultaneously, the highest level of positive epistasis could save plasmids from extinction. But, it depended on a high conjugation rate and on plasmid interactions that further increased conjugative transfer. Both epistasis and the intracellular interaction acting on the conjugative transfer of the focal plasmid X were shown to be asymmetric. This means that positive effects enhancing fitness and transfer had more determinant impacts on plasmid fate than negative effects that impaired those traits with an opposite or greater strength.

Among the conjugative interactions, α_X_ was the most critical for the survival of plasmid X, because the transfer of this plasmid increased when the competitor plasmid was present in the same cell. In such a case, the interaction α_*y*_ was less important as it targeted the competitor plasmid. Notwithstanding, one should consider these interactions also in the last set of simulations where the two plasmids can interact in multiple ways. In such cases, α_X_ = 10 was necessary for the survival of the focal plasmid X but also of the competitor plasmid Y. Therefore, if analyzed from the perspective of plasmid Y, the importance of α_X_ and α_*y*_ would be reversed. It would be preferable to increase the transfer of its competitor than its own. The reason is that this would favor the emergence of XY cells since γ_X_ > γ_*y*_. Nonetheless, the intracellular interaction on conjugation would still be more determinant than the intercellular.

From our models, the four conditions required for plasmid survival were: not too high fitness cost coupled with positive epistasis and, high conjugation transfer coupled with an intracellular effect further increasing it. Indeed, positive epistasis has been pointed out as an explanation for the existence of combinations of small and big (mobile) plasmids (San Millan et al., 2014a). On the other hand, intracellular interactions between conjugative plasmids decrease transfer rates more often than not. Therefore, plasmid combinations simultaneously enjoying intracellular effects on conjugation and fitness (epistasis) might be rare. Moreover, positive epistasis may result from inhibition (rather than facilitation) of conjugative transfer, such that host fitness increases by saving resources that would be spent on the expression of the conjugative machinery (Chao et al., 2000). Studies that can reveal a linkage between epistasis and intracellular interactions on conjugation, or the lack of it, are important to understand how interference between plasmids determine their stability in bacterial populations.

As proof of concept, our models show that interactions between plasmids can enhance their stability. Nevertheless, we acknowledge some limitations. To reduce the number of simulations we considered both plasmids X and Y to have equal loss probabilities, which is unrealistic. Additionally, our simulations encompass long times but consider static features, such that the entities are immutable (neither plasmids nor bacteria change by mutation or by recombination). Therefore, we neglect compensatory mutations that could mitigate the fitness cost of one (San Millan et al., 2015) or several plasmids (Loftie-Eaton et al., 2017; Hall et al., 2020; Jordt et al., 2020) and recombination events between plasmids allowing acquisition of addiction systems that alter loss rates (Loftie-Eaton et al., 2016; Stalder et al., 2017), pleiotropic effects between mutations (Jordt et al., 2020) or even the formation of co-integrates. The latter is especially important from a clinical point of view, in particular upon a fusion of plasmids encoding different resistance mechanisms thus creating multidrug-resistance plasmids (Garcia et al., 2007; Desmet et al., 2018), or even between resistance and virulence plasmids (Dong et al., 2018), that could explain the positive correlation between the diversity of resistance and virulence genes across metagenomes (Escudeiro et al., 2019). We also only considered interactions between two plasmids, but interactions between multiple plasmids (Gama et al., 2017c) can be considered in the future. Despite the limitations of our models, they demonstrate the contribution of interactions between plasmids, and their relative roles, for plasmid survival and should pave the way for further studies with more complex models.

## Data Availability Statement

The code, datasets and analyses presented in this study can be found in online repositories. The names of the repository/repositories and accession number(s) can be found below: https://github.com/GAMMAtri/simulations_plasmid_interactions.

## Author Contributions

JG, RZ, and FD conceived the study. JG and FD designed the experiments. JG performed the experiments. JG and FD analyzed the data. JG wrote the first draft of the manuscript, with contributions of RZ and FD. All authors contributed to manuscript revision, read and approved the submitted version.

## Conflict of Interest

The authors declare that the research was conducted in the absence of any commercial or financial relationships that could be construed as a potential conflict of interest.
